# Ankyloblepharon filiforme adnatum in a preterm neonate: a case report highlighting early recognition and surgical management

**DOI:** 10.1093/jscr/rjaf810

**Published:** 2025-10-08

**Authors:** Abdulmajeed Al Khathami, Renad S Al Subaie, Manal Al Subaie, Faisal Ali A Alsalouli, Khamiso Khan

**Affiliations:** Department Of Ophthalmology, King Fahad Hospital, Al Baha Health Cluster, Al Baha, P.O. 12157, Al Baha, Saudi Arabia; College of Medicine, King Faisal University, Al-Ahsa, Saudi Arabia; College of Medicine, University of Bisha, Bisha, Saudi Arabia; College of Medicine, University of Bisha, Bisha, Saudi Arabia; Department Of Ophthalmology, King Fahad Hospital, Al Baha Health Cluster, Al Baha, P.O. 12157, Al Baha, Saudi Arabia

**Keywords:** ankyloblepharon filiforme adnatum, congenital eyelid fusion, preterm neonate, early surgical management, ocular anomaly

## Abstract

Ankyloblepharon filiforme adnatum (AFA) is a rare congenital anomaly characterized by fine bands of tissue connecting the upper and lower eyelids, potentially obstructing vision if left untreated. We report a case of a preterm male neonate born at 33 weeks and 4 days of gestation via emergency cesarean section due to maternal preeclampsia. On examination, the infant had bilateral eyelid fusion consistent with AFA, confirmed by ophthalmologic evaluation. Surgical division of the adhesions was performed under general anesthesia with no complications. Postoperative recovery was uneventful, and follow-up confirmed clear corneas and normal ocular structures. This case highlights the importance of early recognition and prompt surgical management of AFA to prevent visual deprivation, particularly in preterm infants. It also underscores the need to assess for possible syndromic associations, although this case appeared isolated. Awareness of AFA among neonatologists and ophthalmologists can support timely diagnosis and intervention.

## Introduction

Ankyloblepharon filiforme adnatum (AFA) is a rare congenital anomaly characterized by the presence of one or more fine, elastic bands of tissue connecting the upper and lower eyelid margins at birth [1]. These filamentous adhesions partially fuse the eyelids at the level of the grey line, resulting in a narrowed palpebral fissure and restricted eyelid mobility. The term AFA specifically refers to this pattern of fine connective bands and serves to distinguish it from complete or partial full-thickness eyelid fusion, which is termed ankyloblepharon [1]. During normal fetal development, the eyelids remain fused until approximately the fifth month of gestation, after which they gradually begin to separate. Complete separation typically occurs by the seventh month [2]. A failure of this process can result in persistent fusion or adhesions, as seen in AFA, where the connecting tissue is often extensile and pliable, and composed of fibrous tissue with occasional vascular components [2].

AFA may present as an isolated ocular finding or in association with a range of systemic anomalies. It has been documented in conjunction with syndromes such as trisomy 18 (Edwards syndrome), popliteal pterygium syndrome, and ankyloblepharon–ectodermal dysplasia–clefting (AEC) syndrome [3]. In some instances, AFA may occur alongside cleft lip and palate, gastrointestinal malformations, or cardiac anomalies, suggesting a potential syndromic or genetic basis in certain cases [3]. Early identification of AFA is essential, as timely surgical separation of the adhesions is typically straightforward and prevents complications such as occlusion amblyopia. Furthermore, recognizing this anomaly may prompt further evaluation for underlying syndromic diagnoses, highlighting the importance of a thorough neonatal assessment.

## Case presentation

A male neonate was delivered at 33 weeks and 4 days of gestation via emergency cesarean section due to maternal preeclampsia. The pregnancy was booked and antenatally followed, but complicated by a maternal urinary tract infection, with *Escherichia coli* identified on urine culture. The infant was born with a birth weight of 1.57 kg, classifying him as low birth weight.

The neonate cried immediately after delivery, and standard resuscitation measures were carried out. Apgar scores were 8 and 9 at 1 and 5 minutes, respectively. Due to prematurity, low birth weight, and mild tachypnea, he was admitted to the neonatal intensive care unit for supportive care, including non-invasive ventilation (NIV). Initial admission diagnoses included prematurity, respiratory distress syndrome (RDS), rule out sepsis, and bilateral fused eyelids, clinically suspected to be AFA.

On physical examination, the neonate appeared active and stable, with mild respiratory distress. Vital signs were as follows: temperature 36.2°C, blood pressure 66/42 mmHg, pulse rate 130 bpm, respiratory rate 59 breaths per minute, and oxygen saturation 97% on NIV. Blood glucose was 2.2 mmol/L. Peripheral perfusion was adequate, and no cyanosis, pallor, or jaundice was observed. A superficial laceration was noted on the left parietal scalp, likely related to delivery, but no other skin abnormalities were identified. Cardiovascular examination revealed normal heart sounds without murmurs. Respiratory examination showed mild tachypnea with equal bilateral air entry and no adventitious sounds. Abdominal examination was unremarkable, with a soft, non-distended abdomen and no palpable organomegaly. Neurologically, the infant was alert with a flat anterior fontanelle and no abnormal movements. Genital examination confirmed a normal male phenotype with both testes descended.

**Figure 1:**
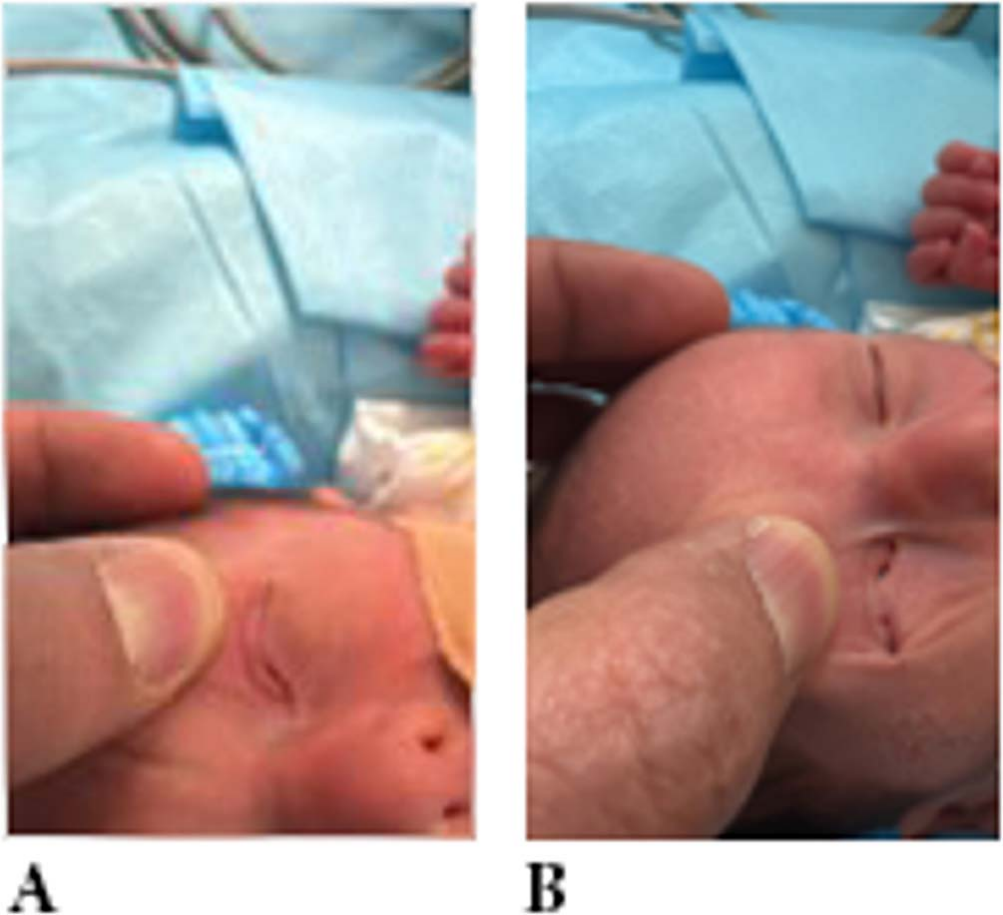
Examination showing eyelid adhesion (ankyloblepharon) in both eyes.

Ophthalmologic evaluation revealed bilateral AFA. Over two-thirds of the upper and lower eyelid margins were joined by fine, extensile tissue bands, completely obscuring the globes bilaterally (Fig. 1). Surgical intervention was planned to release the adhesions under general anesthesia.

## Surgical management

**Figure 2:**
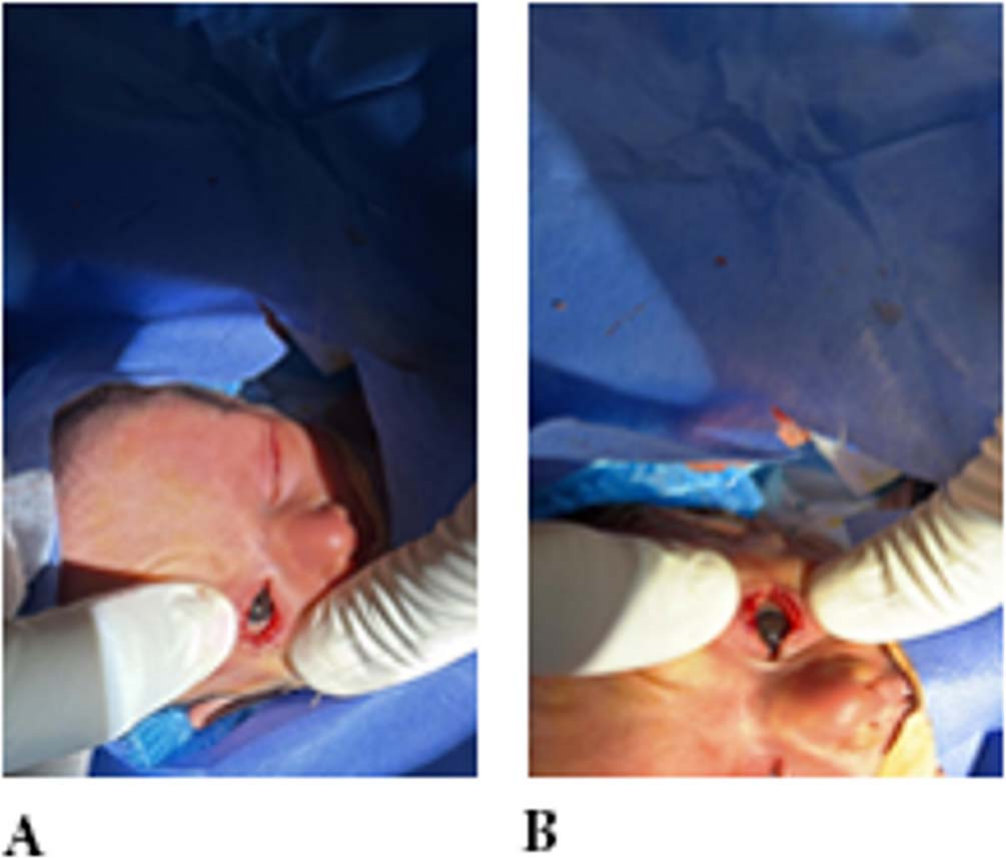
Examination showing eyelid adhesion released surgically in both eyes.

Under general anesthesia, the fibrous bands were carefully divided using fine scissors. A viscoelastic agent (Healon) was applied intraoperatively to protect the ocular surface. Minimal bleeding occurred at the cut sites and resolved spontaneously within a few minutes. Following separation, both corneas and lenses were fully visualized and appeared clear bilaterally. Postoperatively, Maxitrol® ophthalmic ointment (neomycin–polymyxin B–dexamethasone) was applied, and both eyes were patched (Fig. 2).

## Postoperative course

**Figure 3:**
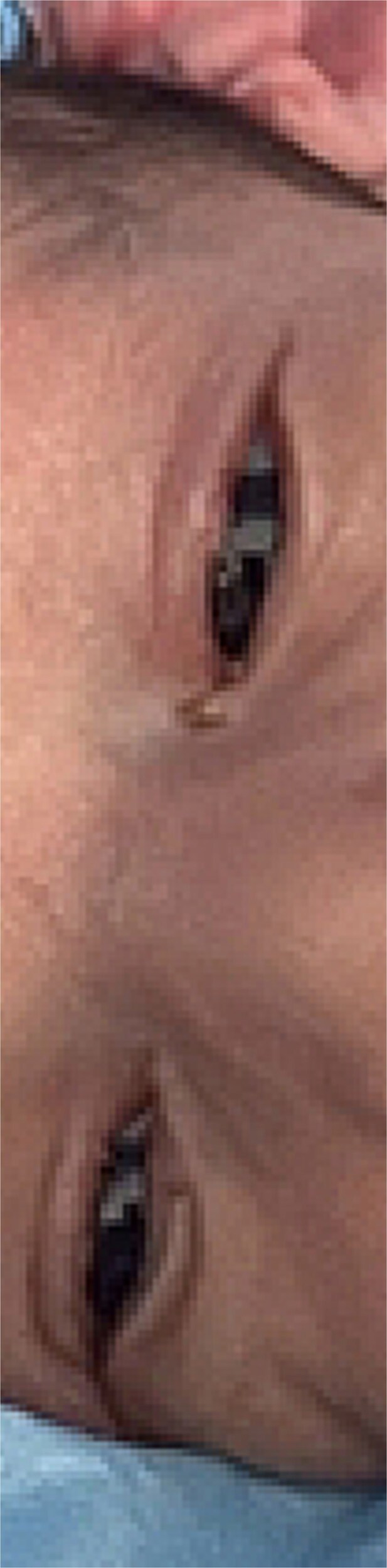
Patient first day post-op showing eyelids freely separated.

On the first postoperative day, the eyelids were fully separated with no residual adhesions (Fig. 3). Corneas remained clear, and Maxitrol® ointment was continued. At one-week follow-up, the pupils were pharmacologically dilated. Bilateral corneas and lenses were clear, and fundus examination revealed a healthy, flat retina with normal optic discs and maculae. There were no signs of recurrent adhesion or other ocular abnormalities.

## Discussion

AFA is a rare congenital condition resulting from the incomplete separation of the eyelid margins during fetal development. Normally, the eyelids fuse early in gestation and begin to separate around the fifth month, with complete separation expected by the seventh month [1]. In AFA, this process is interrupted, leading to the persistence of fine bands of tissue connecting the upper and lower eyelids at the grey line. These adhesions limit eyelid mobility and reduce the palpebral fissure width, potentially interfering with visual development if left untreated [3].

The fibrous strands in AFA are typically extensible and may contain a vascular component. They vary in length from 1 to 10 mm and may present as a single or multiple adhesions, usually at the central or lateral portions of the eyelid margin [4]. Unlike complete ankyloblepharon, which involves full-thickness fusion of the eyelids, AFA is characterized by partial adhesion via filamentous bands and is often amenable to simple surgical release.

AFA may occur as an isolated anomaly or in association with a broad spectrum of congenital disorders. Syndromic associations include trisomy 18 (Edwards syndrome), popliteal pterygium syndrome, and AEC syndrome [4, 5]. It may also accompany other anomalies such as cleft lip and palate, genitourinary malformations, or cardiac defects. Consequently, the presence of AFA should prompt a thorough evaluation to exclude underlying syndromic diagnoses.

The differential diagnosis includes other congenital eyelid fusion anomalies. For example, ankyloblepharon may also result from intrauterine infections or amniotic band syndrome, which are often associated with scarring and other systemic anomalies. Cryptophthalmos, as seen in Fraser syndrome, is a more severe condition characterized by complete eyelid fusion with underlying ocular structures and is typically accompanied by major systemic malformations [5]. Blepharophimosis–ptosis–epicanthus inversus syndrome may resemble AFA due to the narrowed palpebral fissure but lacks the filamentous adhesions [6].

In our case, the neonate was born preterm at 33 weeks and 4 days of gestation following an emergency cesarean section due to maternal preeclampsia. The pregnancy was further complicated by a urinary tract infection with *E. coli*, which may have contributed to fetal stress or disrupted morphogenesis. Although AFA is generally not considered a direct consequence of prematurity, the incomplete eyelid separation could plausibly be related to the gestational age at delivery, as normal separation typically completes by the seventh month.

Prompt recognition of AFA allowed for early ophthalmologic evaluation and surgical management. The procedure involved simple division of the fibrous bands under general anesthesia, with postoperative examination confirming healthy ocular structures and no residual adhesions [7]. Early intervention is critical in preventing deprivation amblyopia, especially in preterm infants who are already at increased risk for retinopathy of prematurity and other visual impairments [1–3, 7].

While our case appears to be isolated and without syndromic features, genetic evaluation may still be warranted given the potential associations with multisystem syndromes. In clinical practice, identifying AFA should serve as a cue for comprehensive neonatal assessment and, when indicated, referral for genetic counseling.

This case highlights both the importance of early detection and the simplicity of effective surgical treatment. It also underscores the need to distinguish AFA from more complex congenital anomalies to ensure timely and appropriate management.

## Conclusion

AFA is a rare but easily recognizable congenital eyelid anomaly that can be effectively managed with timely surgical intervention. In this case, early diagnosis in a preterm neonate enabled prompt and uncomplicated separation of the eyelid adhesions, resulting in full restoration of eyelid function and preservation of ocular integrity. Although AFA is often an isolated finding, its presence warrants a comprehensive clinical evaluation to rule out associated syndromic or systemic anomalies. Clinicians should maintain a high index of suspicion in neonates presenting with fused eyelids, particularly in the context of prematurity or perinatal complications. Early recognition and management are key to preventing potential visual impairment and ensuring optimal developmental outcomes.

## Data Availability

The datasets used and/or analyzed during the current study are available from the corresponding author upon reasonable request.
